# Support Science by Publishing in Scientific Society Journals

**DOI:** 10.1128/mBio.01633-17

**Published:** 2017-09-26

**Authors:** Patrick D. Schloss, Mark Johnston, Arturo Casadevall

**Affiliations:** a Department of Microbiology and Immunology, University of Michigan, Ann Arbor, Michigan, USA; b Department of Biochemistry and Molecular Genetics, University of Colorado Denver, Denver, Colorado, USA; c Department of Molecular Microbiology & Immunology, Johns Hopkins University, Baltimore, Maryland, USA

**Keywords:** journal, publication, societies

## Abstract

Scientific societies provide numerous services to the scientific enterprise, including convening meetings, publishing journals, developing scientific programs, advocating for science, promoting education, providing cohesion and direction for the discipline, and more. For most scientific societies, publishing provides revenues that support these important activities. In recent decades, the proportion of papers on microbiology published in scientific society journals has declined. This is largely due to two competing pressures: authors’ drive to publish in “glam journals”—those with high journal impact factors—and the availability of “mega journals,” which offer speedy publication of articles regardless of their potential impact. The decline in submissions to scientific society journals and the lack of enthusiasm on the part of many scientists to publish in them should be matters of serious concern to all scientists because they impact the service that scientific societies can provide to their members and to science.

## EDITORIAL

The American Society for Microbiology (ASM), which has more than 50,000 members, fulfills its mission to “promote and advance the microbial sciences” (1) by organizing the major microbiology meetings in the United States, publishing 18 scholarly journals, advocating with the public and government for support of microbial sciences, supporting an honorific academy that holds colloquia on emerging topics related to microbiology and public policy, providing a structure for the interaction of microbiologists through its many programs and events, and more. ASM and other scientific societies are nonprofit organizations that support these diverse and important activities through several sources of income: journal subscriptions, member dues, conference proceeds, gifts, and endowment income. None of these sources provides a windfall; most scientific societies depend on all of them to make ends meet.

Microbiologists have many choices of where to publish their work, and new journals appear regularly. When deciding where to submit a paper, most scientists weigh many factors, including editorial expertise, journal scope and audience, publication costs, open access availability, length limitations, prior experience with the journal, reputation in the field, and journal impact factor (JIF). These factors are those that most immediately advance reception of an author’s work and career, but authors should seriously consider the broader impacts of publishing a manuscript in a scientific society journal.

Although scientific scholarly publishing began as an activity of scientific societies to provide visibility and credibility for their discipline (2), the share of papers published in non-scientific-society journals has steadily increased over time and has accelerated in recent years (3). To illustrate these trends in our own field of microbiology, we analyzed papers that are indexed in PubMed and contain microbiology-related key words and that were published in microbiology-specific journals or were published by microbiology professional societies.

**FIG 1:**
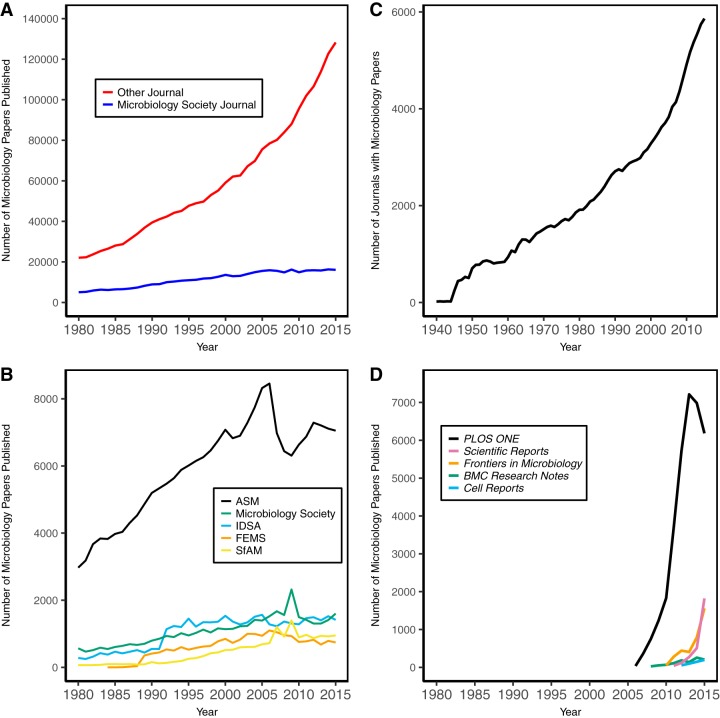
Analysis of publishing trends among microbiology papers. (A) Comparison of the numbers of microbiology papers published in microbiology society journals to those published in all other journals by year. (B) The numbers of microbiology papers published in the five largest microbiology society publishers. (C) The numbers of journals that have published microbiology papers since 1947. FEMS, Federation of European Microbiological Societies; IDSA, Infectious Diseases Society of America; SfAM, *Scientific American*. (D) The numbers of microbiology papers published in mega journals. The data aggregated from PubMed on 22 August 2017 are available from FigShare (https://dx.doi.org/10.6084/m9.figshare.5378425), and the code used to scrape the data from PubMed is available at https://github.com/SchlossLab/Schloss_SocietyJs_mBio_2017.

Microbiology society journals have never published the majority of papers in the discipline, but their growth tracked that of non-microbiology society journals until the early 2000s (Fig. 1A). Part of this has been due to the multidisciplinary nature of the field of microbiology, which supports publishing in a large number of journals. Among the microbiology societies that publish journals, the five that are most highly represented in PubMed include ASM (*n =* 262,282 papers), the Microbiology Society (*n =* 49,556), the Infectious Diseases Society of America (*n =* 44,462), the Federation of European Microbiological Societies (*n =* 21,158), and the Society for Applied Microbiology (*n =* 19,559). As of 2015, these societies published 15, 5, 2, 5, and 5 journals, respectively. Among the 10 journals with the most microbiology papers indexed in PubMed, 7 (*Journal of Bacteriology*, *Journal of Virology*, *Applied and Environmental Microbiology*, *Infection and Immunity*, *Journal of Clinical Microbiology*, *Antimicrobial Agents and Chemotherapy*, and *Journal of Infectious Diseases*) are published by a microbiology professional society, 1 (*Journal of Biological Chemistry*) is published by an allied professional society, and the remaining 2 are *PLoS One* and *Proceedings of the National Academy of Sciences*. It is clear that journals published by professional societies have had a significant impact on the dissemination of microbiology research. What is troubling is that the number of papers published in microbiology society journals has plateaued (Fig. 1B).

Several factors have influenced the number of papers published in scientific society journals. First, the number of journals that publish microbiology research has grown considerably since 1944, when there were 21 such journals; in 2015, there were 5,861 journals that published at least one microbiology paper (Fig. 1C). This proliferation has significantly increased competition between journals, resulting in a thinning of the number of papers being submitted to any one journal. Second, the past 10 years has seen an explosion in the number of papers published in mega journals (4, 5). For microbiology papers, these have most notably included *PLoS One* (launched in 2006), *Scientific Reports* (2011), *Frontiers in Microbiology* (2010), *BMC Research Notes* (2008), and *Cell Reports* (2012) (Fig. 1D). In 2015, mega journals published 9,965 microbiology papers, 6.9% of all microbiology papers. With the exception of *PLoS One*, mega journals are published by for-profit publishers who leverage the growth of these journals to drive their revenues.

Author choices of journals to publish their findings have changed in recent decades. Beginning in the 1980s, scientists, funding agencies, and administrators increasingly assessed the quality of a research article not by characterizing the article and its impact but by transferring a supposed metric of journal quality and impact to all of the articles published in it. The JIF has been widely derided for the lack of transparency in how it is calculated, for focusing on only a 2-year window of citations, for its sensitivity to highly cited papers and to the popularity of subdisciplines (e.g., medicine versus agriculture), and for its focus on the quality of the journal rather than on the quality of individual articles (6, 7). Nevertheless, the JIF remains a major consideration for many scientists in choosing journals for publication of their work. The “glam” journals maintain their high JIFs by limiting their size to create artificial scarcity. In contrast, society journals have a mandate to serve their fields and constituencies by disseminating relevant scientific information that is of high quality regardless of its potential short-term impact. Consequently, society journals publish more papers than the “glam” journals, which dilutes the contribution of highly cited papers to their JIFs, keeping them relatively low.

The proliferation of journal titles has been partly the result of branding of journals affiliated with the prestigious title of a publishing company. Some publishers recognized an opportunity to leverage the high JIF of their flagship journal to create more specialized journals that compete with society journals. For example, in the last 25 years, *Nature* and *Cell* have spawned 40 specialty journals, each of which directly competes with (usually long-standing) society journals. A poignant example of the attraction of the brand name is provided by the numerous scientists who claim that their paper was published in *Nature Scientific Reports*, a journal that does not exist! The journal is actually the Nature Publishing Group mega journal *Scientific Reports*.

This created a tiered system of journals that funnels manuscripts that do not meet the restrictive criteria of the publisher’s flagship journal to their secondary and tertiary journals. In the past, authors who had their paper rejected by *Nature* might have submitted it to a scientific society journal; they now submit it to *Nature Microbiology* and, if rejected there, then to *Nature Communications* and then to *Scientific Reports*. The result has been authors turning away from the society journals that serve the long-term goals of a scientist’s career development and research objectives and toward journals serving the publisher’s goals of maximizing the JIF of the journal and maintaining profits. One result is a vicious downward spiral for scientific society journals because fewer submissions mean fewer highly cited papers, which puts downward pressure on the JIF, leading to even fewer submissions. One can add the decline of society journals as one more unintended consequence of JIF mania.

Changes in the publishing industry over the last 15 years have also put academic journals under stress. The migration to electronic journals required technology and expertise that many publishers were not able to acquire because of insufficient financial resources. Consequently, many society journals contracted with independent, for-profit publishers. For example, *The ISME Journal* (*ISME J*) is a journal of the International Society for Microbial Ecology that is published by Springer Nature. Similarly, the Federation of European Microbiology Societies enlisted Wiley-Blackwell, and, more recently, Oxford University Press, as its publisher. The societies benefit from the publisher’s technological expertise; the publisher gains a venerable title and the opportunity to capture rejected manuscripts for its other specialty and mega journals. It remains to be seen how these partnerships will impact the long-term health and editorial independence of society journals.

Scientific societies support their members’ career development through their journals. The peer review process helps scientists develop their writing skills for their own manuscripts. Serving as peer reviewers provides scientists the opportunity to evaluate others’ research and reflect on how best to craft their own stories, structure their manuscripts, and write clearly. Unfortunately, the peer review process is often characterized as an adversarial arms race between authors, reviewers, and editors that does little to significantly improve the original manuscript. Society journals strive to convert an adversarial process into an educational and edifying one. This has been part of the motivation behind the academy track at *mBio* and the expedited review and *mSphereDirect* tracks at *mSphere* (8–10). Editors of society journals strive to help authors write more clearly and to focus the attention of reviewers on fundamental aspects of research rigor rather than on short-term impact.

Just as the prefixes “Nature” and “Cell” seem to bring gravitas to many journal titles for some scientists and represent implied signals for authors and readers about the quality of the papers that they publish, journals published by professional scientific societies should carry the same authority. After all, they have a long tradition of authoritative leadership and management and are edited by some of the most accomplished scientists in their fields. Professional societies provide legitimacy to the journals they publish. When an author submits a paper to a scientific society journal, or when someone reads a paper published in a scientific society journal, they can be assured that the journal is legitimate and has a decades-long track record of quality.

Scientific society journals offer their authors and members considerable value beyond their benefit to authors’ careers and support of the societies’ missions. The journals published by ASM cover all of bacterial, archaeal, eukaryotic, and viral biology, as well as the response of hosts to microorganisms, and they present a broad view of how this knowledge is applied, from discovery research to research applied in diverse fields, including agriculture, environmental science, food production, public health, and medicine. Professional societies such as ASM are in a unique position to effectively disseminate research to policy makers, educators, and the public as part of a cohesive message, something rarely done by independent publishers because it is not part of their mission.

Scientific society journals are managed and edited by scientists actively working in the fields covered by the journals. A journal metric that quantifies the expertise, academic standing, and experience of academic editors is the journal authority factor, a journal metric based on the accomplishments of the editors. It is inversely correlated with the JIF (11). In other words, authors often prefer to publish in independent journals with high JIFs regardless of the expertise or recognition of the editors. Although there are certain challenges in using academic editors (12), they bring the experience, expertise, and authority that enables professional societies to refine their missions and set the standards of their fields as they evolve.

A general societal trend has been a generational shift in how people view the role of civic, volunteer, and professional organizations. If scientists fail to see a benefit from their professional scientific society, then they will feel no need to support its mission through publishing in and working for the society’s journals. That will have very real negative consequences, creating a negative-feedback loop, and will undercut the mission of professional societies. Professional societies provide great value to their members and to society, and a robust publishing program is essential for that. Please support scientific society-sponsored journals. Our scientific enterprise benefits from them.
