# Erratum for Schloss et al., “Support Science by Publishing in Scientific Society Journals”

**DOI:** 10.1128/mBio.01926-17

**Published:** 2017-11-21

**Authors:** Patrick D. Schloss, Mark Johnston, Arturo Casadevall

**Affiliations:** a Department of Microbiology and Immunology, University of Michigan, Ann Arbor, Michigan, USA; b Department of Biochemistry and Molecular Genetics, University of Colorado Denver, Denver, Colorado, USA; c Department of Molecular Microbiology & Immunology, Johns Hopkins University, Baltimore, Maryland, USA

## ERRATUM

Volume 8, no. 5, e01633-17, 2017, https://doi.org/10.1128/mBio.01633-17. There was an error in reference 12. It should appear as shown below.

12. Johnston M. 2017. On using academic peers as editors for scientific journals. Science Editor 39:67–68. https://www.csescienceeditor.org/article/using-academic-peers-editors-scientific-journals/.

